# Using network models in person-centered care in psychiatry: How perspectivism could help to draw boundaries

**DOI:** 10.3389/fpsyt.2022.925187

**Published:** 2022-09-16

**Authors:** Nina S. de Boer, Daniel Kostić, Marcos Ross, Leon de Bruin, Gerrit Glas

**Affiliations:** ^1^Department of Philosophy, Radboud University, Nijmegen, Netherlands; ^2^Institute for Science in Society, Radboud University, Nijmegen, Netherlands; ^3^Department of Philosophy, Faculty of Humanities, Vrije Universiteit Amsterdam, Amsterdam, Netherlands; ^4^Department of Anatomy and Neurosciences, Amsterdam UMC, Amsterdam, Netherlands

**Keywords:** boundary problem, network analysis, person-centered care, personalized models, perspectivism, psychiatry, topological explanation

## Abstract

In this paper, we explore the conceptual problems that arise when using network analysis in person-centered care (PCC) in psychiatry. Personalized network models are potentially helpful tools for PCC, but we argue that using them in psychiatric practice raises *boundary problems*, i.e., problems in demarcating what should and should not be included in the model, which may limit their ability to provide clinically-relevant knowledge. Models can have explanatory and representational boundaries, among others. We argue that perspectival reasoning can make more explicit what questions personalized network models can address in PCC, given their boundaries.

## Introduction

Mental disorders often dominate the lives of people who experience them^1^. In order to understand these conditions, it is crucial to recognize and examine an individual's symptoms, as well as their personal experience and situational context. For instance, an individual's experience may be influenced by *biological* factors, such as fighting an infection, being malnourished or one's microbiome (1); *social* factors such as unemployment and lack of social support, and *psychological* factors such as their personality type and factors that contribute to their resilience. These personal, contextual factors could influence what symptoms someone develops and how they experience their condition^2^.

Despite the recognition that personal and contextual factors play an important role in psychopathology, clinical research has increasingly moved away from focusing on these types of factors. A prime example of this is the impressive proliferation of neuroscientific research in the last three decades, that has given neurobiological factors a privileged explanatory status in psychopathology. Today, it is not uncommon to hear phrases such as “you are your brain” or to encounter headlines like “the (adjective) brain,” where the brackets are filled in with categories like “female/male,” “teenage,” “addicted,” “hyper-active” and so on. This trend is known as *neuroessentialism*: the idea that denotes the brain as the essence of a person, with the brain being synonymous with concepts like the “self” (2). In line with neuroessentialism, the former director of the National Institute of Mental Health (NIMH), Thomas Insel, even claimed that mental disorders are no more than brain disorders (3). Evidently, the brain is a fundamental organ for the mind, which among other things is reflected by the fact that brain damage is associated with impoverished perceptual and cognitive abilities. However, equating mental disorders with brain dysfunction neglects these other personal, contextual factors that play an important role in understanding psychopathology. Moreover, it has been argued that our theories and models serve as heuristic strategies: they help to describe phenomena, to facilitate their prediction and manipulation, and to make them more intelligible (4, 5). Neuroessentialism, as a theory of psychopathology, ignores the web of relationships between an individual and their context that co-determines their identity. By ignoring these aspects of psychopathology, neuroessentialism may actually obscure mental disorders rather than making them more intelligible to clinicians, patients, and researchers.

Accompanying this development, we have seen a decreased emphasis on the subjective aspects of mental disorders. For instance, neuroessentialism implies that neuroscientific data alone provides an exhaustive insight into the objective core of psychopathology, while personal experience is merely a subjective reflection of this fundamental biological core. Hence, according to this view, knowledge about the pathogenesis of disease belongs to the objective core, whereas values, patient interests, and clinical intuitions belong to the soft margins surrounding that core. The separation between objective and subjective aspects of being ill is also related to the birth of evidence-based medicine (EBM). EBM emerged as a new paradigm for clinical care in medicine and psychiatry. It states that psychiatrists should conscientiously, explicitly, and judiciously use the current best scientific evidence in making decisions for patient care (6). EBM created a hierarchy of evidence where meta-analyses of randomized clinical trials were placed at the top, while clinical intuition and personal experience were placed at the bottom. However, both neuroessentialism and EBM are inadequate for diagnosing and treatment of mental health problems, chiefly because these approaches neglect the personal and contextual factors that play an equally important role in mental disorders.

In reaction to these methodological and conceptual shortcomings of neuroessentialism and EBM, person-centered care (PCC) arose as a guiding vision on how to diagnose and treat individuals. PCC has traditionally been used in nursing, especially in geriatrics (7). Its aim is to respectfully care for an individual considering their preferences, needs, and values, and to ensure that these aspects guide all clinical decisions (7, 8). In this way, the alliance between a therapist and a patient is emphasized. Mezzich [(9), p. 335] gives the following description of person-centered medicine, which we think applies well to PCC:

“[A] medicine *of* the person (of the totality of the person's health, including its ill and positive aspects), *for* the person (promoting the fulfillment of the person's life project), *by* the person (with clinicians extending themselves as full human beings, well-grounded on science and with high ethical aspirations) and *with* the person (working respectfully, in collaboration and in an empowering manner through a partnership of patient, family, and clinicians). The person here is conceptualized in a fully contextualized manner.”

What role does scientific evidence play in PCC? PCC does not reject the use of scientific evidence in psychiatry. Rather, it aims to place it in a framework that is sensitive to the patient's experience, context, and personal values (5). However, integrating these personal and contextual factors into a scientific framework is no easy task. How can we use scientific methods in a way that captures PCC's tenets and is fruitful for both patient and therapist? As we will argue in this paper, psychiatry is finding new avenues to do so with the help of recent developments in *network analysis*. However, it is important to consider whether network models that do justice to the person-, context- and value-dependency of mental disorders could provide clinically-relevant knowledge. Indeed, network models could be used to represent almost anything, and making network models personalized and context-sensitive may make decisions on what should or should not be included in the model less principled. This lack of boundaries may limit the epistemic power of such models in clinical practice. In this paper, we examine the epistemic boundaries that arise when using network models as tools for PCC, and address how perspectivism can be used to inform our theorizing on these boundaries. The paper is structured as follows. First, we discuss the network approach to mental disorders in more detail and examine why network models could be used as tools for PCC. Second, we discuss how boundary problems arise when using personalized network models of mental disorders in PCC. Third, we assess what kind of knowledge about mental disorders personalized network models can provide by examining their representational and explanatory boundaries. Fourth, we examine perspectivism and how it can help us demarcate personalized network models. Finally, we address how perspectival reasoning can shed light on the relevant explanation-seeking questions that personalized network models could afford in clinical practice.

## The network approach to mental disorders

What is network analysis, and why could it be used as a tool for PCC? Network analysis is inspired by principles of graph theory, which state that a network is a system whose elements are connected and mathematically represented as a graph. A graph is a set of nodes (variables of the network) and edges (connections between the nodes) (10). The nodes may represent any kind of variable and the edges could represent any kind of connection between them. We can use network analysis to quantify the connectivity patterns in a graph. These mathematically quantifiable connectivity patterns are called *topological properties* (see Box 1 for more information). Network analysis has been applied to numerous fields like telecommunications, economics, city planning, semantics, biology, neuroscience, and social sciences. In the past years, network analysis has also been applied to the study of mental disorders. Indeed, proponents of the network approach to mental disorders [e.g., (19, 20)] argue that mental disorders should be conceptualized as networks of interconnected symptoms. On this approach, non-symptom factors (such as adverse life events, inflammation, abnormal brain functioning, or genetic mutations) are either considered part of the “external field” of factors affecting the symptom network (19) or constitutive of symptoms or symptom-symptom relations (20). So, the network approach to psychopathology provides an alternative means of conceptualizing mental disorders.

Box 1 **A non-exhaustive overview of topological properties that can be used in network psychometrics. A network is a collection of nodes and edges. A node is a variable within a network (e.g., anhedonia could be a node in a symptom network), and an edge is a connection between nodes in a network (e.g., a partial correlation in a psychometric network). In weighted networks, edges can also represent the strength of the (positive or negative) relation.**

**Table:** 

**Nodal (local) properties**	
Path length	The number of edges required to get from one node to another.
Node degree	The sum of edges maintained by a single node.
Betweenness centrality	The relative number of shortest paths between any pair of nodes passing through a node (11). This measure has been taken as an indication of the role that a node plays in information flow or communication in a network.
Closeness centrality	The average shortest distance from a node to all other nodes in a network (12)
Eigenvector centrality	The extent to which a node is connected to central nodes. It is proportional to the sum of the degrees of a node's neighbors (13, 14).
**Cluster properties**	
Local clustering coefficient	The number of pairs of neighbors of a node that are directly connected, divided by the number of potential pairs of nodes in that neighborhood (15).
Community detection	Means of detecting whether a network is subdivided into separate (non-overlapping, interconnected) modules (16).
**Global network properties**	
Global degree	The average sum of edges maintained by the nodes in the network (17).
Network density	The edges that are present in a network, relative to the number of potential edges (18).
Small-worldness	The ratio of clustering coefficient to path length (15). Networks that demonstrate small-worldness are more efficient than randomly connected networks.
Global clustering	The mean of local clustering coefficients (15), and an indication of the network's robustness.

Proponents of the network approach argue that in order to obtain better insight into mental disorders, we should study *symptom networks* empirically. What role could such quantitative network models play in clinical practice? Of course, scientific models are not able to address all questions pertaining to clinical practice: there are many (epistemic) aspects of clinical practice that are not best addressed by scientific models (e.g., tacit knowledge). However, there are various reasons why network models may be suitable scientific tools for clinical practice in general, and for PCC more specifically. First, the network approach to PCC emphasizes that mental disorders involve a multitude of factors instead of one root cause, thereby moving away from reductionistic (neuroessentialist) interpretations. So, network models could be suitable tools for PCC because they promote a multidimensional view of the nature of mental disorders. Also, network models can be construed in ways that do justice to relevant characteristics of an individual, their disorder, and their context. Novel data collection methods allow researchers to obtain such personalized data based on which personalized network models can be estimated. For instance, recent developments in *experience sampling methods* (ESM) (21) allow people to report on their thoughts, feelings, behavior, and environment using apps on their electronic devices. This modern form of ESM is called *ambulatory assessment* (22) and allows researchers to get insight into relevant patterns of someone's daily life. It has been argued that “ESM enables a more detailed understanding of psychiatric phenomenology” which may provide useful information for treatment targets [(23), p. 1534]. Indeed, various studies have investigated whether estimating personalized symptom networks based on ESM data could provide therapists with new insights and tools for treatment [e.g., (24–26)]. The types of personalized network models that are most commonly used are vector-autoregressive (VAR) models. In VAR modeling, networks are based on time series data, in which nodes represent symptoms and the edges denote (partial) correlations between symptoms^3^. VAR models can be used to estimate *temporal networks* (in which edges represent how one variable predicts another at a later measurement window) and *contemporaneous networks* (in which edges represent the partial correlations between variables in the same measurement window after controlling for the other variables in the same measurement window and all variables at the previous measurement window) [for more information on how to estimate and interpret such VAR models, see (27, 28)]. These quantitative models can be construed on the basis of time series data of one person, and could include clinical, physiological and contextual data, amongst others. Hence, whereas many statistical methods rely on larger samples of subjects, these models could be construed on an individual basis. Because of this, the construction of personalized networks could allow for the incorporation of the patient's experiences and values, which may provide better insight into their clinical picture. Therefore, network models, due to their potential to be personalized, could be a tool for PCC.

Another way that network models could be adapted to be in line with the principles of PCC is to add *salutogenic*, or health-promoting factors. Salutogenesis refers to the study of the origins of health (salus is ‘health’ in Latin, genesis is ‘origin’ in Greek) (29). Indeed, salutogenesis is considered one of the principles of PCC: we cannot fully understand someone with a mental disorder diagnosis if we do not consider factors that promote their well-being. As the World Health Organization (WHO) stated almost fifty years ago, health is not merely the absence of disease or infirmity (30). If psychiatric practice and our models of mental disorders only focus on symptom reduction, this implicitly adheres to the definition that health is the absence of disease. Moreover, it has been demonstrated that simply decreasing negative mental states does not necessarily increase positive mental states (31, 32). So, from the perspective of PCC, it makes sense to include health-promoting factors in our models of mental disorders. In fact, various authors have emphasized that we need to have an *open methodology* of what to place in a network model in order to truly capture an individual's condition (33). In line with PCC, it has been suggested that the focus of network models on symptoms and not on health-promoting factors is a missed opportunity (34): there is nothing inherent to network models that poses this limitation, and including them would make sense from a clinical perspective.

Network analysis has already been applied to the study of well-being. For instance, empirical studies have examined the structure of well-being (35), and subjective well-being in autism spectrum disorder (36). However, in line with PCC, it is also possible to integrate health-promoting factors into symptom networks. How can we perform network analysis in such a way that it incorporates and/or does justice to the interrelations between symptoms, contextual influences, and health-promoting promoting factors? This could either be done by simply incorporating these different components as variables into the analysis [e.g., (37)], or by making use of more advanced network analysis methods such as multilayer networks (38) that could do justice to the difference between these psychometric items. These network models could combine the different factors using cross-sectional data. However, in line with the principles of PCC, it is also possible to construct personalized VAR models that incorporate both symptoms, health-promoting factors, and contextual factors (39, 40). However, if we allow network models to be personalized by including health-promoting and other contextual factors, does this not amount to drawing the boundary too broad for clinicians, patients and researchers to make sensible inferences on their basis? Attempts to move beyond symptoms inevitably give rise to questions concerning what factors (not) to include^4^. We will discuss this problem in more detail in the following section.

## Network models: How to draw their boundaries?

**Figure 1:**
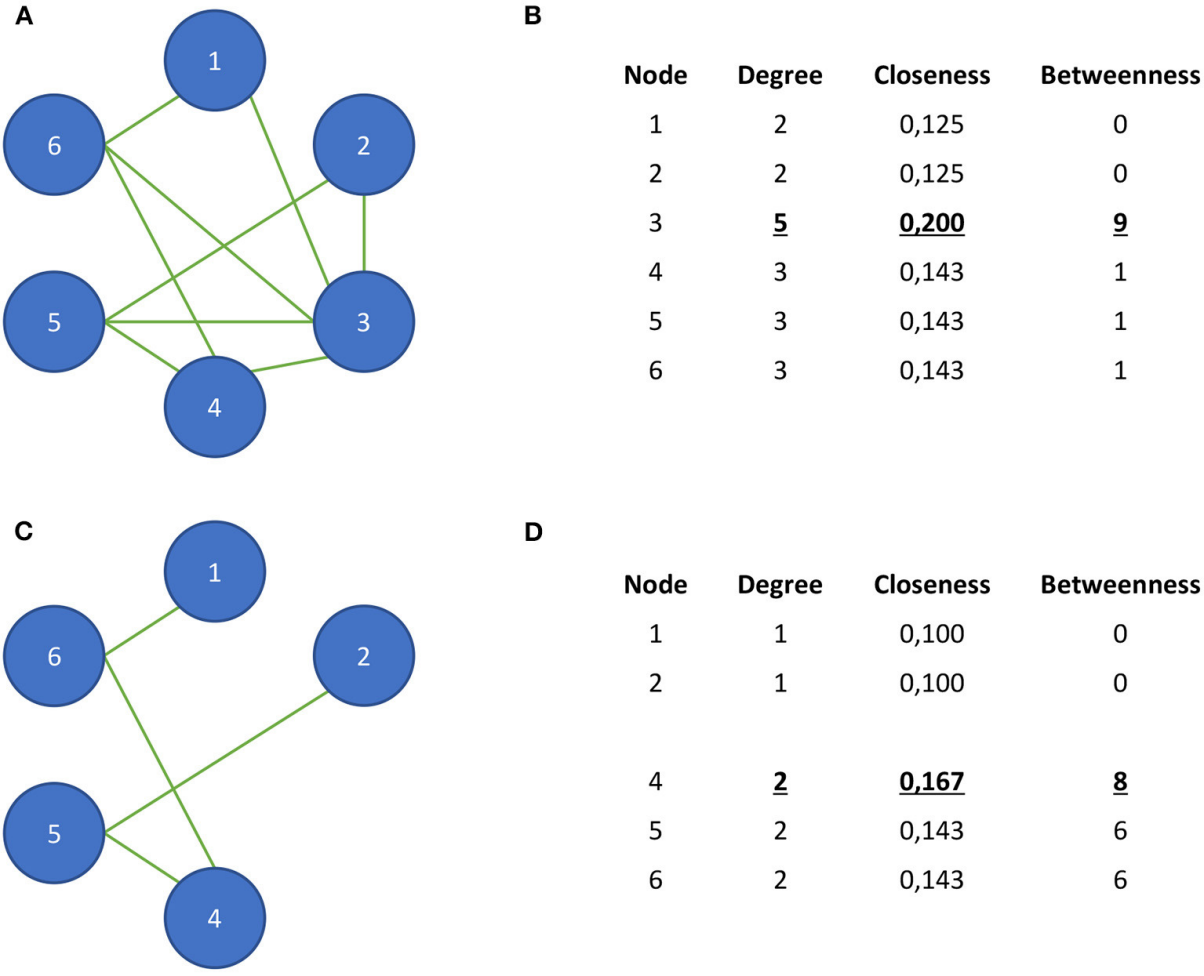
A hypothetical example to illustrate the influence of node selection on local topological properties in a network. In **(A)**, we see a hypothetical network that consists of six nodes. **(B)** demonstrates that node 3 has the highest node degree, closeness centrality, and betweenness centrality. **(C)** shows the same network in which node 3 is removed. **(D)** shows the influence of this removal on the network's centrality measures. Now, nodes 4–6 have the highest node degree, and node 4 has the highest closeness and betweenness centrality. Moreover, the betweenness centrality values of nodes 5 and 6 have strongly increased.

What are the boundaries of network models, and what are the epistemic consequences of how we define the boundary of these models? A boundary, in its most basic definition, is present when an entity is somehow demarcated from something else (42). However, deciding how to demarcate an entity from its surroundings is not always straightforward. Boundary problems arise where there is a lack of consensus or principled reasons for demarcating a system, i.e., deciding what elements we should consider as being part of the system and as being external to it. It has been argued that such difficulties inevitably arise when we deal with phenomena that are constituted or influenced by multiple factors: even physical systems rarely have clearly defined boundaries (43). Why is this an issue for the use of personalized network models in PCC? This problem with system demarcation translates directly to problems in model demarcation. For network models, this means that uncertainties about how to define a system of interest will affect our *node selection*, i.e., selecting the variables that we want to include in our model. This has important implications for the types of explanations, predictions, and knowledge that personalized network models can provide. Node selection can strongly influence the topological properties of network models, impacting the conclusions researchers draw on their basis (44, 45). For instance, the value of the topological measure *betweenness centrality*, i.e., the relative number of shortest paths passing through a specific node (11), is highly influenced by the other nodes that are included in the network (46). This means that removing or including one additional factor in the network can have a great impact on the betweenness centrality values of individual nodes (see Figure 1 for an illustration of this phenomenon). Another reason why node selection is important is that models serve as *epistemic tools* that guide our reasoning about and understanding of the phenomena they represent: they make complex phenomena more intelligible and manageable (47, 48). This is of particular importance in clinical practice, since models can determine how both the therapist and the patient reason about the latter's condition. Hence, where we draw the boundary of personalized network models (i.e., what nodes we select) has important epistemic (and clinical) consequences. How, then, should we decide where to draw the boundary of personalized network models of mental disorders? And how to justify this decision? In the next section, we will examine in more detail how the use of personalized network models could constrain the type of knowledge that these models can provide in clinical practice.

## The representational and explanatory boundaries of personalized network models

What boundaries do personalized network models provide? More specifically, what features of these models constrain the knowledge about mental disorders that we can obtain when using them? Here, we will discuss two types of boundaries that these models afford: representational and explanatory boundaries.

First, the statistical techniques that are used in estimating personalized network models will influence how the network is represented, and hence what kind of interpretations of mental disorders the model affords. These types of boundaries can be referred to as *representational boundaries*, i.e., constraints that are related to the model's representation and its construction. Network representations themselves do not provide many constraints on what can be represented. Network models typically capture global and very abstract features of a system, whereas, for instance, mechanistic models capture more fine-grained and local features (49–57). However, nodes and edges can in principle represent anything. So, it could be argued that network models are representationally *boundless*: they do not provide inherent constraints on what nodes can be included and can be extended indefinitely in size or scale. Having said that, network models in general, and VAR models more specifically, do provide some, albeit limited representational constraints. For instance, VAR models cannot represent how the structural relations between variables will change over time (58), nor how the variables in the network may be related to each other on other timescales. So, making use of VAR models does provide some representational constraints, and thereby influences the type of information that these models can provide.

Relatedly, personalized network models seem limited to providing only certain types of explanations. *Explanatory boundaries* concern the constraints provided by the types of explanations that a particular model can provide. It is commonly agreed that models in general (59–61), and network models of mental disorders in particular (62) have an *exploratory* function: they can be used as exploratory tools for estimating potential network structures from psychological data, or as methods to generate hypotheses about the development and treatment of mental disorders. However, network models of mental disorders may also provide explanations. What types of explanations of mental disorders could personalized network models provide? The first possibility is that these models provide *topological explanations*, i.e., explanations that are based on the topological properties of a network. We argue that this is the most promising explanatory potential of these models because network models in general are particularly suited to provide such explanations (49, 52, 55–57, 63–66). What criteria should personalized network models of mental disorders meet in order to provide topological explanations? As argued by (54), this requires that the topological properties and empirical properties that feature in the model are approximately true, and also stand in an appropriate counterfactual dependence relation to each other (this will be discussed in more detail in later sections). Second, could personalized network models provide *mechanistic explanations*? Mechanistic explanations show how the working parts of a phenomenon that are organized into a mechanism either cause a phenomenon of interest or constitute a phenomenon that is at a higher level (67, 68). Some philosophers have argued that if network models provide any explanation at all, it is a mechanistic one (69). According to this view, mechanistic explanations show how the working parts that are organized into a mechanism either cause the phenomenon of interest or constitute a phenomenon that is at a higher level (think of how the macro-physical property of hardness is constituted by the micro-physical atomic structures). Given this, personalized network models will not provide mechanistic explanations if any of the following mechanistic conditions are violated: (1) nodes and edges in a network model denote working parts of a mechanism, (2) the explanandum (what is to be explained) is at a higher level than the explanans (what does the explaining), and, (3) topological properties are causally responsible for the explanandum (57). Since the nodes and edges in personalized network models will likely violate conditions 1 and 3, they do not provide mechanistic explanations. More precisely, the first condition is violated because the time series and correlations between them that are represented in VAR models are not spatiotemporal working parts of a mechanism (they are merely conventional). The third, causal responsibility condition is violated because the topological properties that are explanatory in VAR models do not precede the phenomenon they explain (they are simultaneous). Since causation requires that causes precede their effects, it is not justified to claim that topological properties in these VAR models cause mental disorders. So, it is unlikely that VAR models can provide mechanistic explanations.

Finally, are personalized network models able to provide *causal explanations*? On the one hand, it has been argued that the edges in the temporal network provide temporal predictions or Granger causality (70), which can be considered an approximation or potential indication of causal relations. However, it is unclear whether VAR models of mental disorders can provide causal explanations (71). For instance, it is unlikely that these models will satisfy interventionist criteria for causality (41, 57, 72). So, whereas personalized network models could provide topological explanations, it is less clear whether they provide mechanistic or causal explanations.

Here, we see how making use of personalized network models provides representational and explanatory constraints, and thereby limits the type of knowledge that these models can provide. To what extent do these considerations inform node selection? Arguably, the boundaries do not only constrain the type of explanations of mental disorders we can obtain based on personalized network models: they also constrain the model itself, i.e., what factors we decide to include. Indeed, the explanatory potential of network models depends on what nodes and edges represent (69). As aforementioned, the explanatory power of personalized network models will depend on whether the topological and empirical properties in question are approximately true (65), which is not limited to representational accuracy of nodes and edges, but also includes justification of particular measurement approaches that are used to obtain and analyse data (73). Hence, if we want personalized network models to provide explanations, this may constrain node selection. However, to what extent will this consideration inform node selection in clinical practice? Assessing these criteria is often difficult in clinical settings, and they do not give us information on what *kind of factors* the model should include. In the next section, we argue that perspectivism could help us provide such constraints on node selection in PCC.

## Perspectivism

As we already discussed, PCC affords certain aims, values, and goals for the therapist and the patient. Here, we argue that it is justified that such perspectival considerations influence node selection. Perspectivism is a philosophical position that emphasizes the importance of perspective-dependent factors in (scientific) theorizing and inquiry. It acknowledges that we cannot study the world in a way that is independent of our own perspective, and that each system can be characterized by multiple perspectives (74). Perspectivism presupposes that our theories and models serve specific goals of interest. They each have a limited range, so the ones that researchers will use depend on their research questions and goals at hand. Hence, perspectivism allows for—and even promotes—the use of a plethora of diverse models to examine complex phenomena, such as mental disorders. In other words, it could be argued that perspectivism promotes explanatory pluralism.

It makes sense to examine personalized network models in light of perspectivism. Indeed, clinical practice is inherently perspectival, and PCC brings its perspectival character to the fore. From a PCC perspective, symptoms are no longer the central focus, but the individual, their coping with the disorder and everything that comes along with it. They can enter clinical practice with various goals in mind: feeling better, functioning better, improving their agency, and finding the right balance between dependence and independence (of help). Moreover, these clinical goals serve as a guide for the questions that the patient and therapist want to address, given a particular individual with a particular disorder in a particular context. For instance, “How can I feel better?,” “How can I function better (in different domains of functioning)?,” “What can I do myself in order to improve my condition?” and “What kind of help do I need?” Hence, in order to be suitable for clinical practice, network models should help us to address these perspectival goals and questions.

These perspectival considerations can also play an important role in deciding what nodes should be included in personalized network models. If we want clinical goals to constrain our node selection, the nodes included should be (1) of relevance to the patient and their situational context, (2) able to guide treatment, and/or (3) able to monitor clinical development. This means that node selection will be determined by the specific problem that the patient wants to address—as decided in collaboration with the therapist—or the symptoms they consider most burdensome (73). For instance, if it is hypothesized that someone's depressive symptoms may be aggravated by their stressful job, this factor should be included in the model. It may also limit nodes to ones on which could be intervened (75), or to items that are most relevant in monitoring whether treatments are effective (76), or in predicting the risk of relapse (77). Moreover, various authors have emphasized that constructing network models of mental disorders should be informed by clearly defined research questions (and hypotheses) that are of personal and clinical relevance (73, 78, 79). So, the clinical setting from which we start our inquiry can provide constraints on node selection.

Does this mean, however, that any variable can in principle be included in personalized network models as long as it is of relevance to the patient and clinician? A general worry is that perspectivism invokes relativism by making node selection too dependent on contingent factors: the inquirer's background knowledge, preferences, or contingent facts about personal circumstances (80–83). One may argue that if this is the case, this may limit the robustness of personalized network models and hence their ability to provide useful knowledge about a patient's condition. This issue is even more pressing if we take personal and contextual factors into account, as would be advocated by PCC. One means by which we could ensure that our models provide knowledge is by being clearer about the clinical questions that personalized network models would actually be able to address. In other words, we should ensure that the clinical questions we want personalized network models to address at least do justice to their representational and explanatory boundaries. In the next section, we will explore how *perspectival reasoning* could help with that.

## Perspectival reasoning and topological explanation in personalized network models

How can we get more insight into the clinical questions that personalized network models could help us answer? To illustrate how this can be done, we can use insights from perspectival (or *erotetic*) reasoning. According to perspectival reasoning, questions can be conclusions in arguments. More specifically, perspectival reasoning demonstrates how we can logically derive questions from sets of propositions (which may include hypotheses) about a model, and empirical observations (84, 85). So, we can start from a set of propositions and derive relevant questions based on the syntax (structure) and semantics (meaning) of those statements. To illustrate this, we can use a toy example inspired by Wiśniewski (2, 85):

(1) If Mary writes three books in one year, then she is a nun, single, or she has a very patient partner.
(2) Mary writes three books in one year.
(3) Is Mary a nun, single, or does she have a very patient partner?

This example demonstrates that we can derive a relevant question—and space of possible answers to that question—by observing what is the case (Mary writes three books in one year), and by keeping in mind the possible explanations of what is the case (she either is a nun, single or has a patient partner). Whilst perspectival reasoning cannot help us to determine the answer to this question, it does make it clear what questions are sensible to ask given the available knowledge^5^.

How could perspectival reasoning be of use for our case at hand, i.e., determining what knowledge personalized network models could provide in PCC? We argue that perspectival reasoning allows us to formulate relevant *explanation-seeking* questions. To illustrate this claim, we will focus on the topological explanatory potential of these models.

What criteria should be met before personalized network models are able to provide topological explanations? We already discussed this briefly in a previous section, but here we will explore this in more detail using the account of topological explanations developed by Kostić (54, 56, 57). Kostić's account provides necessary and sufficient conditions under which a network model provides a genuine topological explanation and does so by explicitly incorporating perspectival criteria. Kostić formulates his account as follows:

*a*'s being *F* topologically explains why *a* is *G* if and only if:
(T1) *a* is *F* (where *F* is a topological property);
(T2) *a* is *G* (where *G* is an empirical property);
(T3) Had *a* been *F'* (rather than *F*), then *a* would have been *G'* (rather than *G*);
(T4) *a* is *F* is an answer to the question why is *a, G*?

What do these criteria entail? T1 states that a system should have a certain network connectivity pattern, expressed as a topological property (see Box 1 for examples of topological properties). T2 states that a system should have an empirical property, e.g., it displays certain behavior. T3 describes the counterfactual dependence between a system's topological and empirical property: the behavior of the system should depend on the presence of the topological property. Topological explanations hence concern a counterfactual dependence. However, if we combine these three criteria, there is still something missing: we do not yet know based on these criteria whether the topological property is an answer to the relevant explanation-seeking question. That is why Kostić's account provides the perspectival criterion T4: in order for a topological property to be an explanation of an empirical property, it should be an answer to the relevant explanation-seeking question. This shows how asking the relevant questions makes it intelligible why some empirical property *G* counterfactually depends on a network connectivity pattern, which is expressed as its topological property *F*.

Let us now apply these considerations to an example that is relevant for the use of personalized network models in PCC. Various studies have examined the global topological property *network density* in personalized symptom networks to predict whether someone is vulnerable to developing (or relapsing into) a mental disorder. In line with the idea that mental disorders behave like complex dynamic systems (71, 91, 92), it is supposed that we are complex systems that may shift from a healthy into a disordered state following perturbations to the system. Perturbations to the healthy state may not have any effects until a tipping point is reached and the system (abruptly) shifts to a disordered state. Researchers have suggested that an increase in symptom network density (i.e., the strength of associations between symptoms) may predict this transition from a healthy to a disordered state (93, 94). This hypothesis has been examined in simulation studies (92) and in small samples of time-series data of individuals with a major depressive disorder diagnosis (93, 95). Hence, if someone has a symptom network that is more strongly connected, they are more likely to develop a mental disorder.

We can use Kostić's scheme to formulate what criteria should be met before we can claim that a strongly connected symptom network can serve as an explanation for this vulnerability. Here, *a* refers to an individual, *F* refers to high symptom network density, and *G* refers to being vulnerable to developing a mental disorder (i.e., entering a disordered state). Hence, the example can be unpacked as follows:

An individual *a* having high symptom network density explains why they are vulnerable to developing a mental disorder if and only if:
(T1) an individual *a* has a high symptom network density (which is topological property *F* in the schema above);
(T2) an individual *a* is vulnerable to developing a mental disorder (which is an empirical property *G* in the schema above).
(T3) had an individual *a* had a low symptom network density (rather than a high symptom network density), then the individual *a* would not have been vulnerable to developing a mental disorder.
(T4) an individual *a* having a high symptom network density is the relevant answer to the question “Why is *a* vulnerable to developing a mental disorder?”

How can we examine whether T4 is the case by making use of the principles of perspectival reasoning? By assessing whether being vulnerable to developing a mental disorder counterfactually depends on high symptom network density, and combining this with the observation that an individual is in fact vulnerable to developing a mental disorder. However, starting with a statement about what it is for an individual to be vulnerable to developing a mental disorder, and the empirical finding that the individual is in fact more vulnerable to developing a mental disorder, we can also come up with a relevant explanation-seeking question. The argument itself provides a space of possible answers. It makes it intelligible why appealing to a dependency between network density and vulnerability counts as an explanation of why the mental disorder has developed (with a particular collection of symptoms). It also makes it intelligible why appealing to different topological properties or even to non-topological properties does not: they are not included in the space of possible answers (96). Here, we can see how the principles of perspectival reasoning can help in dealing with the boundaries of personalized network models in clinical practice: it can help derive questions that are epistemically fruitful for both explanatory and clinical purposes. It also suggests that we should limit our personalized network models to nodes about which we have specific (topological) hypotheses.

## Conclusion

In this paper, we have provided a conceptual analysis of the boundary problems that arise when using personalized network models in PCC. PCC focuses on individuals and considers mental disorders as highly context-dependent. There are various aspects of network models that make them suitable as tools for PCC, including their ability to be personalized by making use of ESM data and their ability to accommodate a variety of different personal and/or contextual factors. However, the type of knowledge that these models can provide for clinical practice is influenced by how we draw models' boundaries. We have argued that the use of personalized network models influences the interpretations and explanations of mental disorders that we can provide. Perspectivism can help to determine what nodes should be included in the model, and perspectival reasoning can help to make the explanations that these models could provide more intelligible.

Using personalized network models in PCC will inevitably invoke problems in node selection and model demarcation. However, our analysis has shown that we can justify our decisions on what factors (not) to include, although this does not mean that the use of network models in PCC is straightforward. One of the important issues in this application is how to determine the *relevance* of the patterns that are found. Moreover, the relevance that a therapist attributes to a pattern may differ from the relevance that a patient attributes to it, for both stakeholders may have different values attributed to these findings. Clinical practice is messy, and there will not be a one-on-one translation of our proposal into clinical guidelines. However, our account may suffice as an example of how network demarcation could work in practice. At last, our account emphasizes the importance of making a patient's context and clinical goals explicit, for this may constrain the range of relevant why-questions that personalized network models could address and could guide these in the right direction.

## Author contributions

Introduction: GG, LB, MR, and NB. The network approach to mental disorders: MR and NB. Network models - How to draw their boundaries, the representational and explanatory boundaries of personalized network models, and perspectivism: DK and NB. Perspectival reasoning: DK, LB, and NB. Conclusion: DK, GG, and NB. Coordination: DK and LB. All authors contributed to the conception, design of the article, and approved the submitted version.

## Funding

DK would like to acknowledge funding from the Radboud Excellence Initiative.

## Conflict of interest

The authors declare that the research was conducted in the absence of any commercial or financial relationships that could be construed as a potential conflict of interest.

## Publisher's note

All claims expressed in this article are solely those of the authors and do not necessarily represent those of their affiliated organizations, or those of the publisher, the editors and the reviewers. Any product that may be evaluated in this article, or claim that may be made by its manufacturer, is not guaranteed or endorsed by the publisher.

## Footnotes

^1^Throughout this article, we will use the term ‘patient’ (“the one who suffers”) to refer to people who seek therapy for their mental health problems. We are aware that the use of this term is contested by some who have been given a mental health diagnosis. For instance, some argue that the term ‘client’ better reflects their experiences. However, each term comes with its own advantages and disadvantages, and the term patient is most suited for the setting that we want to address (i.e., psychiatric, clinical practice). Similarly, we will use the term ‘mental disorder’ to refer to the mental health problems that people experience and are treated for in clinical practice, whilst acknowledging that not everyone who has been diagnosed will resonate with this term.

^2^The sociocultural and historical context in which an individual operates also plays an important role in the diagnosis and treatment of mental disorders. Among others, it influences what is considered pathological. To illustrate, homosexuality was considered a mental disorder by the Diagnostic and Statistical Manual of Mental Disorders (DSM) until 1973. Fortunately, homosexuality is not in the DSM anymore, but it is likely that the disease classification will have influenced people's conception of their homosexuality in the past. However, quantifying sociocultural and historical influences in scientific models is far from straightforward. Hence, these factors will not be discussed explicitly in the remainder of the article (we would like to thank one of the reviewers for putting this point forward).

^3^VAR models should not be confused with dynamical system models, which are based on sets of differential equations and may provide directed (causal) relations between variables (e.g., causal loop diagrams). So, it is important to note that the claims we make with respect to the epistemic potential and boundaries of VAR models do not necessarily extend to dynamical system models.

^4^The appearance of a boundary issue when including environmental factors in network accounts of psychopathology has already been emphasized by de Boer et al. [(41), 6]. It is important to note that the epistemic issues related to system and model demarcation, are not specific to psychopathology and/or network models. In fact, as one of the reviewers pointed out, boundary issues may be widespread in modeling practices. However, we argue that the specific questions concerning system/model demarcation and the consequences it bears, will differ per model and context in which the model is used. Hence, in this article, we focus on how boundary problems play out with respect to personalized network models in PCC.

^5^This example differs from more familiar examples of deductive arguments in two ways. First, whereas traditional deductive arguments derive a conclusion which is also a proposition, this argument derives a question. Second, perspectival reasoning requires a disjunction of hypothetical propositions in the first premise, where any of the disjuncts could be true. The second premise specifies more closely what is the case. And based on that we are able to derive a relevant question, which also implies a space of possible answers. For the technical details of the logic of this type of arguments, see (86–90).
